# Effects of Open-Label Placebos on State Anxiety and Glucocorticoid Stress Responses

**DOI:** 10.3390/brainsci11040508

**Published:** 2021-04-16

**Authors:** Michael Schaefer, Julian Hellmann-Regen, Sören Enge

**Affiliations:** 1 Medical School Berlin, Calandrellistr, 1-9, 12447 Berlin, Germany; soeren.enge@medicalschool-berlin.de; 2 Section Clinical Neurobiology, Department of Psychiatry and Psychotherapy, Campus Benjamin Franklin, Berlin Institute of Health, Charité—Universitätsmedizin Berlin, Corporate Member of Freie Universität Berlin, Humboldt-Universität zu Berlin, 12203 Berlin, Germany; julian.hellmann@charite.de

**Keywords:** open-label placebos, placebos, stress, MAST, cortisol, anxiety

## Abstract

Stress belongs to the most frequent negative feelings people are confronted with in daily life. Strategies against acute stress include, e.g., relaxation techniques or medications, but it is also known that placebos can successfully reduce negative emotional stress. While it is widely held that placebos require deception to provoke a response, recent studies demonstrate intriguing evidence that placebos may work even without concealment (e.g., against anxiety or pain). Most of these studies are based on self-report questionnaires and do not include physiological measures. Here we report results of a study examining whether placebos without deception reduce acute stress. A total of 53 healthy individuals received either placebos without deception or no pills before participating in a laboratory stress test (Maastricht Acute Stress Test, MAST). We recorded self-report stress measures and cortisol responses before and after the MAST. Results showed no significant differences between the placebo and the control group, but when comparing participants with high relative to low beliefs in the power of placebos we found significant lower anxiety and cortisol responses for the placebo believers. These results show that non-deceptive placebos may successfully reduce acute anxiety and stress, but only in participants who had a strong belief in placebos. We discuss the results by suggesting that open-label placebos might be a possible treatment to reduce stress at least for some individuals.

## 1. Introduction

When we are exposed to a threatening situation, our brain activates two physiological systems in order to cope with this stressful event, the autonomic nervous system (ANS) and the hypothalamic–pituitary–adrenal (HPA) axes. While the ANS stimulates the secretion of adrenalin and noradrenaline (resulting in increased arousal and attention), the activation of the HPA system results in releasing glucocorticoid cortisol [1,2]. The role of these two systems varies depending on the intensity and type of stress (for example, psychological vs. physiological stress). Both systems are important, since they result in cognitive and behavioral adaptation that helps to face the psychological or physiological threat.

There are different strategies to cope with stressful events, including, for example, cognitive–behavioral skills training, yoga, massage, mindfulness-based interventions, but also medication [3,4,5,6]. Medications may be antidepressants or, to reduce acute stress, benzodiazepines or other GABA (Gamma aminobutyric acid)-like substances, which are known to be linked to the risk of addiction [7].

It has also been demonstrated that placebos reduce symptoms associated with (acute) stress. For example, Darragh et al. found that a take-home placebo treatment successfully reduced stress and anxiety symptoms in a non-patient population [8]. Furmark et al. demonstrated a placebo effect on stress-related activity (stressful public speaking task) in the amygdala [9]. Balodis et al. examined stress-reducing placebo effects using the Trier Social Stress Test and found that both placebo and alcohol made participants (self)report less tension and anxiety, accompanied by a smaller increase in cortisol [10]. Using an fMRI approach, Petrovic et al. employed placebos to manipulate expectations and reported an effect on the perception of emotionally unpleasant pictures and associated brain activation [11].

Furthermore, studies have shown that placebos can modulate neuroendocrine functions and immune responses both in rodents and humans [12,13,14]. For example, Goebel et al. reported conditioning effects of immunosuppression in humans [4]. The authors paired the immunosuppressive drug cyclosporine A (unconditioned stimulus) with a gustatory stimulus (conditioned stimulus). Subsequently, the mere exposition to the conditioned (the gustatory) stimulus resulted in a modulation of the immune system typical for cyclosporine A.

Given that prescribing a placebo can be considered as a psychological intervention, it is also interesting that numerous studies have shown that psychological interventions can modulate the immune response [15]. Early studies reported how hypnosis affects immune responses [16]. More recent approaches used cognitive–behavioral therapy or relaxation and mediation as psychological interventions. Furthermore, psychological interventions based on the patient’s expectation, social support, or psychoeducation have also been reported to affect stress related responses [17,18,19,20]. For example, it has been shown that in human immunodeficiency virus (HIV)-positive patients, psychological interventions are efficacious in modulating HIV disease markers such as hormone regulation and immune status [21]. However, there are also studies pointing to the lack of effects in disease progression based on these psychological interventions (e.g., [22]).

Until very recently, placebos were always administered in a deceptive way. Some new studies challenge this requirement and demonstrate that non-deceptive or open-label placebos (OLPs) can reduce symptoms in many clinical disorders such as pain, migraine, irritable bowel syndrome, depression, anxiety, ADHD (attention-defict/hyperactivity disorder), or allergic symptoms (e.g., [23,24,25,26,27,28,29,30,31]). However, since prescribing deceptive placebos is linked to severe practical and ethical problems (e.g., undermining informed consent and trust), OLPs might be a promising new way to overcome these difficulties [32,33].

Recent studies have shown that OLPs may also help people with non-clinical problems. For example, it has been demonstrated that OLPs improve exercise performance in cyclists [34]. Furthermore, we recently reported reduced test anxiety and improved self-management abilities in students when receiving OLPs [35]. Test anxiety and self-management skills in this study were assessed by self-report measures, similar to other OLP studies. However, given that self-report measures may be prone to response bias, it remains unclear whether the beneficial effects reported by this and other OLP studies are based on psychobiological effects. Only very few studies on OLPs so far include biological measures, often with mixed results (e.g., [36]).

The current study tries to address this issue and examines whether OLPs may reduce both self-reported stress symptoms and psychobiological measures associated with stress. Since it has been shown that acute stress is responding to deceptive placebos [11], we hypothesized that stress symptoms may also be reduced by OLPs. This is in particular supported by recent work on the impact of OLPs on anxiety relief in academic test situations [35].

In order to test our hypothesis, we conducted a study with two groups. While the OLP group received placebos for three weeks, the control group did not get any pills. Subsequently, we invited all participants to a laboratory stress test. We used the Maastricht acute stress test (MAST), an established and validated measure to induce stress [37]. The MAST consists of an acute stress phase including exposures to cold pressor trials and mental arithmetic calculations with negative feedback to increase social pressure. In addition to self-report measures to felt stress we also collected neuroendocrine stress markers before and in response to the MAST (salivary cortisol levels). Based on previous work (e.g., [11,35], we hypothesized that OLPs may reduce both self-reported and biological measures of stress more than a control group.

## 2. Materials and Methods

### 2.1. Participants

We recruited 60 German-speaking students with no neurological or psychiatric history via advertisements in flyers, social media posts, and at the local university. Seven individuals were excluded due to use of corticosteroid medications (3) or experiencing no stress in the MAST (e.g., because of knowing the task), resulting in 53 participants (mean age 26.43 ± 9.09 years, 28 females). All participants gave written informed consent to the study, which adhered to the Declaration of Helsinki. The participants received course credit for their participation. The study was approved by the ethical board of the German Psychological Society (Deutsche Gesellschaft für Psychologie, DGPs).

Sample size was determined with respect to previous studies [38]. Using an estimated effect size of d = 0.8 (with alpha error probability 0.05) our calculation resulted in in 26 participants per cell as necessary for a desired power of 0.80.

### 2.2. Maastricht Acute Stress Test

The MAST is an established procedure to induce stress in a laboratory context and was conducted according to a previously published protocol (see [37]). According to Smeets et al. the procedure started with a preparation phase in which the experimenter explained the task (about 5 min). In the following acute stress phase, the participants had to put their right hand into cold ice-water (2 °C) in 5 trials (each lasting from 60 to 90 s). In between the hand immersion trials participants were engaged with mental arithmetic calculations. They were asked to count backwards starting at 2032 in steps of 17. Whenever the participants made a mistake or counted too slow, they received negative feedback and had to start again at 2043. This acute stress task lasted for 10 min. In order to further increase social-evaluative pressure, participants are told that they will be video-taped. For further details see Smeets et al. [37].

### 2.3. Neuroendocrine Stress Responses

Saliva samples were collected at five time points using Salivettes (Sarstedt, Nümbrecht, Germany) for determination of salivary cortisol levels and stored at −20 °C.

**Figure 1:**
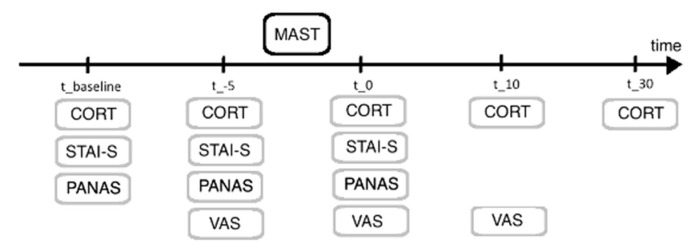
Overview of experimental procedure and timeline. Both groups participated in the identical procedure. VAS = visual analogue scale, STAI-S = State-Anxiety-Inventory, PANAS = positive and negative affective scale, MAST = Maastricht acute stress test, CORT = cortisol sample.

Participants provided first saliva samples 15 min after arriving (35 min before the MAST) and 5 min before MAST (baseline and pre-stress:t_baseline, t_−5). The following 3 saliva samples were taken immediately after the MAST (t_0), 10 min (t_10) and 30 min after the MAST (t_30) (see Figure 1). After completion of the study, all samples were transferred to the Neurobiology Laboratory of the Department of Psychiatry, Charité—Universitätsmedizin Berlin, Campus Benjamin Franklin, where all biochemical analyses were performed. For cortisol determination, the saliva samples were thawed and centrifuged at 2000× *g* for 10 min. Salivary free cortisol levels were determined using an adapted homogenous time-resolved fluorescence resonance energy transfer (HTR-FRET)-based competitive immunoassay (Cisbio/PerkinElmer, Codolet, France) as previously published [39,40,41]. To ensure a reliable assay performance, authentic standards for standard curve preparation and positive control samples were prepared and included in each assay run. Authentic standards have been cross-validated using an independent competitive immunoassay (IBL, Hamburg, Germany). Intra-assay coefficients of variation were below 8%, inter-assay coefficients of variation were below 10% for cortisol measurements. The lower limit of detection was below 0.2 nmol/L.

### 2.4. Procedure

Participants were told that the study was about psychophysiological interactions and stress. After signing written informed consent participants provided sociodemographic and clinical/psychological data (i.e., age, gender, BMI, sport activities, smoking status, alcohol use, corticosteroid medication, hormonal contraception) via self-report. Any type of corticosteroid medication resulted in an exclusion of study participation. After providing sociodemographic data we asked the participants to complete questionnaires with respect to their experienced chronic stress level and personality. Chronic stress was measured with the 12-item screening questionnaire (SSCS) of the Trier Inventory of Chronic Stress (TICS; [42]) measuring chronic stress of the past three months on a 5-point Likert scale ranging from 0 (“never”) to 4 (“very often”). The SSCS demonstrated a good reliability and validity [42]. Internal consistency (Cronbach’s alpha) of the scale in the present sample was 0.90.

The five-factor model of personality was measured using the German version of the Big Five Inventory 2, which shows good psychometric characteristics (BFI-2, [43]). The BFI-2 comprises 60 items that have to be rated on a 5-point scale ranging from “do not agree at all” to “completely agree” to tap the broad personality traits extraversion, neuroticism, openness, conscientiousness and agreeableness. Internal consistency (Cronbach’s alpha) of the scales in the present study ranged from 0.60 to 0.89.

In addition, we asked the participants about their belief and expectations in placebos. This short questionnaire included 4 statements such as “Placebo effects can occur in all illnesses and conditions”. Participants indicated their response using a Likert-scale (1 = strongly disagree, 6 = strongly agree). The items were used to build a composite score for the belief in placebos. The questionnaire is identical to Leibowitz et al. [44].

After completing the questionnaires, all participants were briefed in the same way on placebo effects. They were explained that placebos are inactive substances and that they contain no medications, but both deceptive as well as non-deceptive placebos may still be powerful [25]. We further explained to them that a possible mechanism for placebo response may be classical conditioning; similar to Pavlov’s dogs that salivated when they heard the bell. In addition, we told them that a positive attitude may be helpful for the placebo effect but is not necessary. Finally, we stressed that taking the placebo pills faithfully is important. These four statements were taken from Kaptchuk et al. and similar studies on OLPs [25,26,29].

The participants were then randomized into two groups by choosing a sealed opaque envelope with the assignment of being in the placebo or in the control group (similar to [35]). Participants in the placebo group received a white tube containing 42 placebo pills. The pills were white, round, and the size was about 4 mm. Placebo pills contained sugar, wheat- and cornstarch, and glucose syrup. The tube with the pills was labeled with the logo of the university and the following information—“Placebo pills (42), take one in the morning and one before night, for 21 days”. Participants in the control group received no pills. We reminded those participants of the importance of the control condition and stressed not to miss the second appointment. All participants (OLP and control group) were also asked to refrain from eating, drinking, smoking, and brushing teeth and to avoid strenuous physical activity at least 1 h prior to the lab session.

After three weeks we invited all participants for a second appointment in our lab, taking place between 1 to 5 pm. Here we again asked the participants to complete questionnaires on chronic stress (SSCS) and on current stress experiences. The last measure employed a visual analogue scale (VAS) consisting of a 100 mm long straight line ranging from 0 (“do not agree at all”) to 100 (“completely agree/absolutely”) on which the participants were asked to rate the subjectively perceived stressfulness using the question ‘the situation was stressful to me’ [45]. Furthermore, subjective stress and anxiety was assessed by using the State-Trait-Anxiety-Inventory (STAI, [46,47]). The STAI is a widely known instrument to measure two distinct concepts of anxiety, trait (STAI-T) and state (STAI-S) anxiety. Both the trait as well as the state scale include 20 statements, which has to be rated on a 4-point scale ranging from 1 “not at all” to 4 “very much so”. The trait scale asks how individuals generally feel, whereas the state scale refers to the feelings of a particular moment. We here used the state dimension to measure actual levels of anxiety due to stressful events. The trait scale was employed in particular to determine any baseline differences of the two groups. In addition, mood and subjective stress was measured by using a German version of the Positive and Negative Affect Scale (PANAS, [48,49]. The PANAS includes 10 markers of positive affective states and 10 markers of negative affective states, which have to be rated on a 5-point scale (1 = very slightly or not at all, 5 = extremely). Higher scores on the negative scale point to experienced negative affect.

After completing the questionnaires, participants started with the MAST, which was followed again by VAS, PANAS and STAI-S questionnaires. Before and after the MAST, cortisol responses were collected (see Figure 1 for a timeline).

### 2.5. Statistical Analysis

We used repeated measures ANOVA with time as a within-subject variable and group (placebo vs. control) as a between-subjects variable using self-reported stress ratings (VAS scores, STAI-S, PANAS, respectively) and belief and expectations in placebos as dependent variables. An analogue procedure was calculated for salivary cortisol levels. Differences in baseline scores (in particular with respect to chronic stress, SSCS) were tested using *t*-tests for independent samples.

## 3. Results

### 3.1. Stress Responses: Self-Report Measures between OLP and Control Group

The OLP and control group were not different with respect to perceived chronic stress and baseline scores (VAS, STAI-S, STAI-T, PANAS, SCSS; *p* > 0.10; see Table 1). Furthermore, the graphs did not differ according to age, BMI, and educational background, sport activities, or smoking status. However, the control group stated they drank more alcohol than the placebo group (*p* < 0.05).

**Figure 2:**
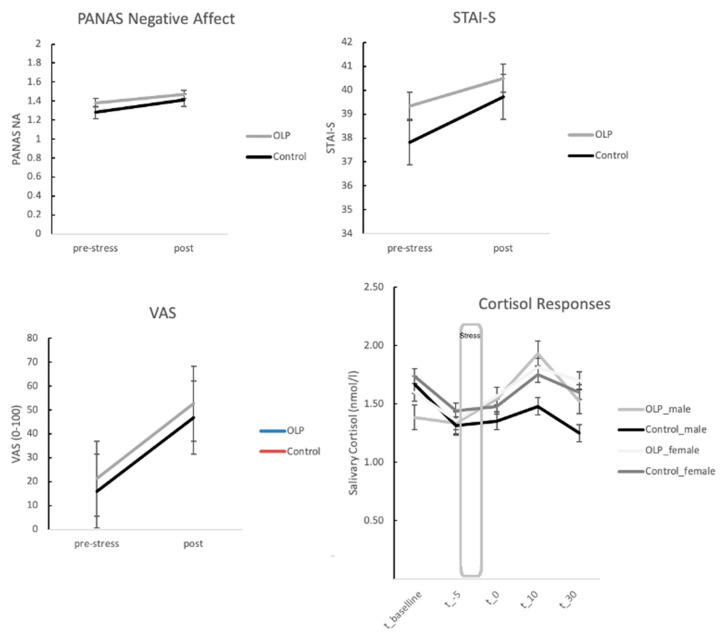
Stress and cortisol responses for the open-label placebo (OLP) and control group (error bars represent standard errors). See text for further details.

Figure 2 shows that the increase of subjective stress responses in STAI-S and PANAS is less pronounced in the OLP group, but this interaction failed to reach the level of significance (see also Table 2). An ANOVA (three time points, t_baseline, t_-5, t_0) for stress and anxiety measured with the STAI-S showed an effect for time (F(2, 102) = 3.00, *p* = 0.066, Greenhouse Geisser correction), but no interaction with group (F(2, 102) = 0.41, *p* > 0.10). PANAS scores for mood and anxiety increased during stress for both groups but failed to reach the level of significance (no main effect or interaction, *p* > 0.10).

Results of a repeated measures ANOVA with factor time (three time points—t_−5, t_0, t_10) and group (OLP vs. control) for perceived stress marked on the VAS revealed a strong main effect for time (F(2, 102) = 27.24, *p* < 0.001), indicating that the stress manipulation was successful, but no interaction with the group (F(2, 102) = 0.58, *p* > 0.10).

### 3.2. Stress Responses: Neuroendocrine Responses between OLP and Control Group

We calculated a repeated measures ANOVA (factors time and group) on mean cortisol concentrations (log transformed). Results revealed a significant main effect for time (again demonstrating successful stress manipulation), but no significant interaction of this effect with group (main effect time—F(4, 204) = 5.74, *p* = 0.007; interaction with group—F(4, 204) = 2.47, *p* > 0.10, Greenhouse Geisser correction) (see Figure 2).

### 3.3. Stress Responses for OLP Group Only: Self-Report Measures for Stress Depending on High vs. Low Belief

In order to further examine the OLP group, we tested whether participants’ belief in the power of placebos may have affected stress-related responses. To this end we divided the OLP group into participants with very strong and less strong beliefs in placebos (median, mean of high belief group—5.08 ± 0.56, *n* = 16, mean of low belief group 3.97 ± 0.34, *n* = 8). Baseline scores of VAS, STAI-S, STAI-T, PANAS, and cortisol responses were not different (*p* > 0.10).

A repeated measures ANOVA on self-reported stress (VAS) with time (three time points) as within and belief group as a between-subjects factor (high vs. low placebo belief) replicated the significant main effect on time. We did not find a significant interaction for self-reported stress scores with respect to high vs. low placebo belief (main effect time—F(2, 44) = 12.06, *p* < 0.001; interaction—F(2, 44) = 0.91, *p* > 0.10).

Separate comparisons between the placebo and the control group for placebo believers only (and low believers, respectively) showed no results with respect to VAS scores (all *p* > 0.10) (control group, high believers—*n* = 18, low believers—*n* = 11).

Repeated measures ANOVA with factor time (three time points) and group for the STAI-S scores revealed trends for a main effect and an interaction with group (main effect—F(2, 44) = 2.57, *p* = 0.088; interaction—F(2, 44) = 2.61, *p* = 0.085). Post hoc t-tests showed that the STAI-S scores increased in particular for the low placebo believer, whereas the scores for the high placebo believer remained stable (low belief group, means pre—38.38 ± 10.98, post—42.13 ± 10.36; high belief group, pre—39.81 ± 5.88, post—39.69 ± 5.04; *t*-test for change scores: t(22) = −1.80, *p* = 0.043; pre scores were not different, all *p* > 0.10). An ANVOA with difference scores of the STAI (pre to post) as the dependent variable and pre-scores as a covariate revealed similar results (F(1, 21) = 3.07, *p* = 0.094). Analogue calculations for the control group (split in high vs. low placebo belief) revealed no significant interaction (*p* > 0.10). Furthermore, an ANOVA with factors time, belief in placebos, and group (OLP and controls) showed no main effect for belief (F(1, 49) = 0.25, *p* > 0.10), but confirmed our findings by demonstrating an interaction between STAI scores, group, and belief (F(2, 98) = 4.86, *p* = 0.014). A main effect for time suggests that stress manipulation was successful (F(2, 102) = 3.00, *p* = 0.066). When comparing the placebo and the control group only for the placebo believers, results showed higher STAI scores for the control group (STAI change scores of controls; 2.89 ± 7.81, t(32) = −3.01, *p* = 0.09). Comparison of the low believers revealed no effects (*p* > 0.10). Thus, placebos seem to affect anxiety feelings compared with the control group, but only for placebo believers.

**Figure 3:**
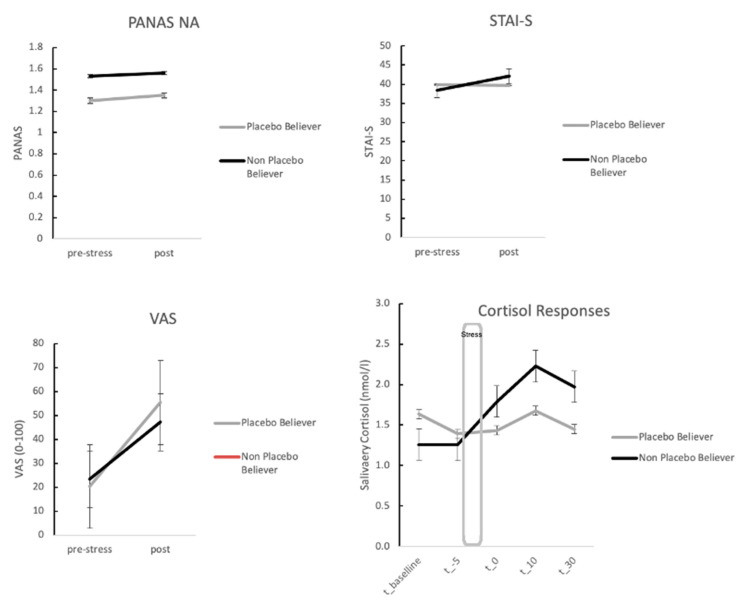
Stress and cortisol responses for high and low placebo believers of the OLP group (error bars represent standard errors). Results demonstrate lower increases for STAI-S scores and cortisol responses in the placebo believer group.

PANAS scores for negative affect increased during stress for both groups but failed to reach the level of significance (no main effect or interaction, *p* > 0.10) (see Figure 3). However, when comparing the placebo and the control group for the placebo believers only, results showed higher PANAS change scores for the control group, but again this difference failed to reach the level of significance (PANAS change scores of believers in placebo group—0.05 ± 0.45; PANAS change scores of believers in controls—0.20 ± 0.45, *p* > 0.10). Comparison of the low believers revealed no effects (*p* > 0.10). An ANOVA with factors, time, belief in placebos, and group (OLP and controls) showed no effects (all *p* > 0.10).

### 3.4. Stress Responses for OLP Group Only: Neuroendocrine Measures Depending on High vs. Low Belief in Placebos

We then calculated repeated measures ANOVA with time (five measurements) and group (high vs. low placebo belief) for cortisol responses. Results replicated the main effect for time (indicating that participants felt stress), but also revealed an interaction between time and placebo group (high vs. low believers) (main effect time—F(4, 88) = 5.62, *p* = 0.013; interaction—F(4, 88) = 4.14, *p* = 0.033) (see Figure 3).

Post hoc t-tests from t_−5 to t_10 demonstrated higher cortisol responses for the low belief group (mean cortisol response low belief group, pre t_−5: 1.25 ± 0.83, post t_10—2.23 ± 1.32, high belief—1.39 ± 0.44, post—1.68 ± 0.60; *t*-test for change scores—t(22) = −1.86, *p* = 0.038; pre scores were not different, all *p* > 0.10). An ANVOA with difference scores as the dependent variable and pre-scores as a covariate revealed similar results (F(1, 21) = 3.00, *p* = 0.098). Analogue calculations for the control group (split in high vs. low placebo belief) revealed no significant interaction (*p* > 0.10).

An ANOVA with factors, time, belief in placebos, and group (OLP and controls) showed no main effect for belief (F(1, 49) = 0.14, *p* > 0.10), but revealed an interaction between cortisol responses, group, and belief (F(4, 196) = 6.66, *p* = 0.03). Furthermore, a main effect for time showed that participants felt stress during the MAST (F(4, 196) = 6.73, *p* < 0.001). Comparisons between the placebo and the control group only for the placebo believers showed no results (*p* > 0.10), but groups of low believers showed significant higher cortisol responses for the placebo group (*p* < 0.05).

### 3.5. Correlations of Personality Measures with Belief in Placebos

**Figure 4:**
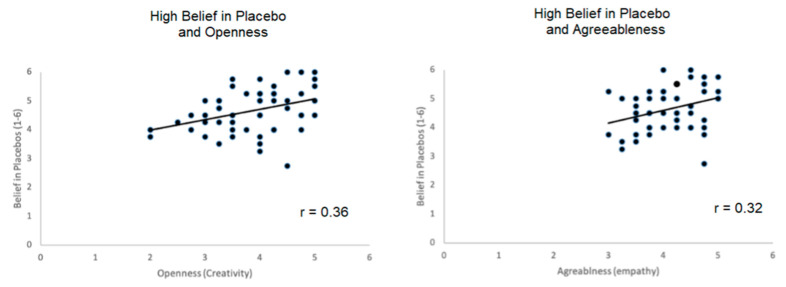
Scatterplot of belief in placebos scores and personality dimensions agreeableness and openness. Other personality dimensions failed to show significant correlations with high beliefs in placebos.

To better characterize the nature of placebo believers, we then computed correlations of personality measures with the belief in the power of placebos. Results showed significant correlations between the personality measure agreeableness and belief in placebos (r = 0.30, *p* = 0.033), in particular with the subscore empathy (r = 0.32, *p* = 0.019) (see Figure 4). In addition, we found a significant correlation of belief in placebos with openness (subscore creativity) (r = 0.36, *p* = 0.009). No other personality dimension showed significant correlations with belief in placebos (extraversion—r = 0.01, neuroticism—r = −0.13, conscientiousness—r = −0.05; all *p* > 0.10). Thus, high believers in placebos are characterized by higher openness and agreeableness scores.

## 4. Discussion

This study aimed to test whether open-label placebos affect the experience of acute stress. Our results did not show any significant differences between the placebo and control group but demonstrated that individuals in the OLP group with a high belief in placebos expressed less anxiety and lower stress-related cortisol responses than participants with low beliefs.

An increasing body of evidence suggests that placebos may have effects even if we know that the pills that we are swallowing are placebos. Most of these studies use self-reported measures to examine these effects. Given that self-assessment ratings may be prone to response bias, physiological measures are desirable to further examine the impact of OLPs. The current study tried to address this question. We employed the MAST to manipulate stress in a laboratory context. Significant increases of self-report measures of stress and cortisol responses demonstrated that the MAST successfully induced stress responses in our participants. The OLP group showed lower increases in anxiety and mood, but these differences failed to reach the level of significance. Although we did not find significant effects between the groups, results revealed that individuals of the OLP group with a high belief in placebos showed less self-reported stress and cortisol responses than participants with a low belief. Thus, OLPs seem to work in this laboratory stress test, but only in individuals with a high belief in the power of placebos. This result is in line with a previous study on symptoms in an allergic symptom paradigm. Using the identical questionnaire to identify participants with a high belief in placebos, Leibowitz et al. [44] demonstrated that OLPs reduced physiological effects for participants who strongly believe in placebos. High interindividual variations when examining effects of OLPs have also been reported in other studies (e.g., [34]).

So, what kind of personality is sensitive to the OLP effect? Both our study as well as the Leibowitz et al. study used the same questionnaire to identify individuals with a high belief in placebos. This questionnaire asks for the general belief in placebos. In our study, almost all of our participants agreed with the statements. Thus, when splitting our participants in two groups, both of them believed in placebos, but the high belief group expressed very strongly their agreement to the statements. When correlating self-reported placebo belief, we found significant positive correlations with agreeableness (in particular the subscore empathy) and openness dimensions of the Big Five, which is in line with recent research on placebo responders [50] arguing that such traits may increase the awareness to inner experiences. Thus, these personality traits seem to make placebo interventions especially promising. In addition, and in view of the items defining the creativity/creative imagination facet of the BFI-2 openness dimension, individuals with higher scores tend to think outside the box and are more open to unconventional ideas [51], which could make them more inclined to believe in the potential effects of placebos. This may also count for more agreeable individuals (empathy subscale), who according to the BFI-2 are described to be sensitive and empathic, which may render them more receptive to believe in the possible impact of placebos on thoughts, feelings and behavior. However, agreeable individuals are generally more compliant and might show a stronger adherence behavior to the study regime, which could have partly contributed to the correlation pattern.

Previous studies already addressed the role of personality factors in placebo responses and pointed to dispositional optimism and low anxiety as predictors for placebo (and nocebo) responses (e.g., [52,53,54]). Furthermore, contextual factors such as narratives, instructions, positive expectations, and interactions with healthcare providers are known to interact with an individual patient’s characteristics, which may also include genetics as well as the experienced medical history [9,55,56]. The present results on personality factors in an OLP study extend these studies.

While we tried to describe the personality of individuals with a strong belief in the power of placebos, it is still unclear why those individuals believe that pills without any active ingredients have any utility. Based on our findings, one could speculate that at the beginning of a placebo treatment, placebo believers are very open-minded and do not think so much about the fact that they swallow an inert substance (in contrast to individuals skeptical to placebos). Then unconscious processes may start, triggered by situational settings, events, or people (e.g., healthcare professionals) and lead to the “automatic activation of internal mental representations and processes” [57], which in turn may cause the beneficial effects of (open-label) placebos. Recent theoretical models pointing to embodied cognition, the Bayesian brain, or the model of prediction and error processing as possible explanations for the placebo effect seem to reflect this enactive perspective (e.g., [33,58]).

Our results revealed that high belief participants in the OLP group showed less self-reported anxiety, supporting previous similar studies on the impact of OLPs on anxiety and stress [39,59]. However, for self-reported measures of stress on VAS scores, we did not find any effects. In the VAS scores participants were asked to rate their stress level in the previous situation simply by putting a mark on a line. The missing effect of OLPs on self-reported stress here might be explained by silent talking of the participants, which has been shown to enable an effortless (without cognitive control) form of self-control [60]. We speculate that these cognitive processes may partly account for missing effects here. In contrast, the STAI-S is a psychological inventory on stress and anxiety, which does not directly ask the participant to rate felt stress. Moreover, the used one-item VAS might assess the underlying construct (acute stress) less accurately than a multi-item measure such as the STAI-S.

Several limitations have to be noted. First of all, the sample size of this study is small, in particular when considering the comparison between high and low placebo believers. Future studies are needed to replicate the results of this pilot study. Furthermore, we did not have a covert placebo group. Thus, we do not know whether the OLP effects are similar to the effects of deceptive placebos in stress. In addition, future studies may also add a control condition of the MAST, which includes a similar procedure, but without inducing stress.

Together with a recent study [59] the current study is one of the first studies demonstrating physiological effects of OLPs along with self-reported measures in acute stress. Guevarra et al. induced stress in an EEG (electroencephalography) study and found reduced stress responses both in behavioral measures as well as in the late positive potential, a biophysiological marker of stress in the EEG. Our results are in line with this outcome, suggesting that stress can be reduced simply by using placebo pills or a placebo description of the intervention prior the acute stress (at least in individuals with high placebo beliefs).

The option to use placebos in order to reduce stress and anxiety seems to be particularly important when thinking of side effects of medications such as addiction of benzodiazepines. However, from a clinical point of view, the general strategy of taking pills (placebo, benzodiazepines, etc.) to reduce stress or anxiety should be considered with caution. While placebos do not have the risks of side effects such as benzodiazepines, for many individuals, counseling or other strategies may be more suitable.

## 5. Conclusions

Taken together, this study demonstrates that OLPs may reduce acute stress and anxiety in participants with a strong belief in placebos. Additional studies are necessary to further understand the way OLPs may work when we feel anxiety and stress. However, we believe that the current results are encouraging to further test whether taking placebos beyond deception might help us to better cope with stress.

## Figures and Tables

**Table 1:** Demographic and baseline characteristics of participants (mean ± standard deviations).

Characteristic	Open Label Placebo	Control
*n*	24	29
age (in years)	25.25 ± 7.28	27.41 ± 10.25
Females/males	14/10	14/15
SSCI	31.13 ± 8.96	29.97 ± 8.26
STAI-T	39.75 ± 8.44	37.31 ± 7.87
STAI-S (baseline)	41.54 ± 7.82	39.31 ± 6.60
PANAS NA (baseline)	1.47 ± 0.65	1.33 ± 0.23
VAS (baseline)	21.33 ± 22.82	16.00 ± 17.69

**Table 2:** Change scores of participants from pre- to post-MAST (mean ± SD).

Self-Report Measures	Open Label Placebo	Control
STAI-S	1.17 ± 5.21	1.90 ± 7.34
PANAS	0.05 ± 0.45	0.12 ± 0.46
VAS	31.29 ± 35.07	30.76 ± 34.60

## Data Availability

The data presented in this study are available on request from the corresponding author.
